# Multi-Level Health Outcomes of Local Food Procurement in United States Farm-to-School Programs: a Systematic Review

**DOI:** 10.1016/j.advnut.2025.100580

**Published:** 2025-12-31

**Authors:** Kathryn E Coakley, Luotao Lin, Diana Gonzales-Pacheco, Olivia M Thompson, Jonathan D Eldredge, Elizabeth Y Jimenez, Melissa Pflugh Prescott

**Affiliations:** 1 College of Population Health, the University of New Mexico, Albuquerque, NM, United States; 2 Nutrition and Dietetic Program, Department of Individual, Family, and Community Education, the University of New Mexico, Albuquerque, NM, United States; 3 Health Sciences Library and Informatics Center, the University of New Mexico, Albuquerque, NM, United States; 4 Department of Nutrition, School of Medicine, Case Western Reserve University, Cleveland, OH, United States

**Keywords:** farm-to-school, farm-to-institution, farms, local food systems, agriculture, schools, school meals, food intake, students, vegetables

## Abstract

Approximately 74% of schools in the United States participated in at least 1 farm-to-school (F2S) activity during the 2022 to 2023 school year. Relationships between specific F2S activities, particularly local food procurement, and health outcomes across multiple levels (individual, family, community, and population) have not been systematically reviewed and reported. We conducted a systematic review of peer-reviewed and gray literature to examine relationships between local food procurement within F2S programs and child, family, producer, and community health outcomes (PROSPERO# CRD420250624067). Secondarily, we cataloged reported economic impacts and barriers and facilitators to local food procurement in F2S programs. Systematic literature searches identified 520 unique records. After title and abstract and full-text screening, 7 peer-reviewed articles and 2 gray literature sources met inclusion criteria, representing 3 cross-sectional, 1 prospective cohort, and 5 quasi-experimental studies. All studies presented individual-level health outcomes and most focused on children’s fruit and vegetable intake. Results suggest local food procurement in F2S programs is associated with increases in children’s vegetable intake, particularly in those with low intake and more intensive F2S exposure, but is not associated with positive changes in fruit intake. Evidence was insufficient to draw conclusions for any other health outcome evaluated at any level. Local food procurement facilitators reported by study authors were program champions, culturally-relevant activities and foods, and family and community engagement. Schools also faced significant barriers related to their ability, capacity, and financial means to source local food. Local food procurement, one of the most common F2S activities in K-12 settings in the United States, is associated with higher vegetable intake among students, but additional rigorous research is needed to determine comprehensive multi-level impacts on student, family, producer, and community health.

**Statement of Significance**

Local food procurement as part of F2S programs is associated with higher vegetable intake but may not be associated with positive changes in fruit intake among children. Additional rigorous research is required to comprehensively assess multi-level impacts on other child, family, producer, and community health outcomes.

## Introduction

Farm-to-school (F2S) programs provide a mix of classroom-based and experiential education activities, local food procurement, and local food promotion to primary and secondary (K-12) school children. Although school gardens have existed since the early 1900s in the United States, the concept of F2S emerged in the late 1990s [1], gaining momentum with the introduction of the USDA Fresh Fruit and Vegetable Program in 2002. The Fresh Fruit and Vegetable Program offers free fresh fruit and vegetable snacks and short nutrition lessons to children at eligible elementary schools [2,3]. In 2010, Congress created the F2S Program, providing funding, training, and technical assistance to support public, nonprofit, private, and charter schools that operate the National School Lunch Program and/or School Breakfast Program in accessing local food [1]. Participation in F2S programs has expanded substantially ever since. Currently, Child Nutrition Program operators and other entities can apply for competitive grants from the renamed “Patrick Leahy Farm to School Program” to strengthen local food systems through activities such as local food procurement and agricultural education [4].

According to the most recent USDA Farm to School Census, in the 2022 to 2023 school year, nearly three-fourths of United States K-12 schools (74%), representing approximately 45.6 million students, participated in F2S programs [5]. As of August 2025, 42 states have signed agreements for the USDA Local Food for Schools Cooperative Agreement Program [6]. Local Food for Schools Agreements award funds to states to assist with purchasing domestic local foods (i.e., food that is grown, raised, caught, or processed within the United States state or within 400 miles of the delivery destination) for K-12 schools and childcare institutions [6,7]. F2S programs also rely on other federal, state, community, and district or school-level funding [5].

F2S programs are tailored to address the specific needs of their community and vary by geographic location, funding levels, and school size and preference. Many F2S program include multiple activities such as serving local foods to students (i.e., using local foods in any form in school meals, taste testing activities, and cooking classes); sourcing and procuring local food (i.e., sourcing from the USDA Department of Defense Fresh Program, local distributors, food producers or producer collectives, processors or manufacturers, or food hubs); providing agricultural or nutrition education; implementing school gardens; and promoting local foods through marketing, events, field trips, and family and community engagement events [5]. In the 2022 to 2023 school year, 68% of schools that participated in F2S reported 5 or more distinct activities, the most common of which was serving local foods (63% of schools), followed by local food sourcing and procurement (55% of schools) [5].

Procuring and serving local foods are common and critical components of F2S programs that shape the types of foods available, enable other F2S activities, and have the potential to impact health outcomes at multiple levels, defined as individual, family/organization, community, and population by the Nutrition Health Disparities Framework [8]. Locally grown fruits and vegetables (F&V) are often perceived as fresher and more nutritious and represent a major component of schools’ local food purchases, potentially leading to increases in participating children’s F&V intake [5]. In the 2022 to 2023 school year, for example, 6 of the top 10 items purchased by schools participating in F2S were fruits or vegetables (apples, lettuce, salad mix, watermelon, oranges, and strawberries) [5]. Increased F&V intake may improve children’s overall diet quality, displace other less healthy food options, and reduce the risk of obesity [[9], [10], [11], [12]]. Improvements in children’s dietary intake at school may extend to the home, not only improving children’s diet quality outside of school, but also impacting the family. Local food procurement in F2S also contributes to local food systems and economies by providing food producers with expanded and predictable markets, generating additional revenue, building relationships within local food systems, and expanding collaborations between schools, producers, and community organizations [13,14]. These F2S benefits could also contribute to improvements in producer and community physical and mental health.

The extant literature largely focuses on relationships between F2S program exposure and student outcomes, including willingness to try new F&V and F&V intake; food selection; knowledge and attitudes about local foods, nutrition, and health; and school meal participation [2,[15], [16], [17], [18]]. Evidence suggests positive relationships, but few studies have detailed specific aspects of the F2S programs studied, such as local food procurement. Moreover, most studies focus on F2S outcomes related to students’ knowledge and perceptions rather than health outcomes (e.g., weight status, mental health). A single scoping review of 21 studies found local food procurement in primary and secondary schools had a positive relationship with students’ F&V intake but did not examine other health outcomes or relationships with outcomes at levels beyond the individual child, such as the family, producer, or community [17].

This systematic review of peer-reviewed and gray literature aimed to fill knowledge gaps by identifying and synthesizing findings from studies examining relationships between exposure to local food procurement within F2S programs and child, family, producer, and community health. These levels were selected based on the Nutrition Health Disparities Framework [8]. Secondarily, we sought to examine barriers and facilitators to local food procurement in F2S programs, along with economic impacts.

## Methods

The 2020 PRISMA statement was used to develop the review framework and methods [19]. Peer-reviewed and gray literature were eligible for inclusion in this systematic review. The final systematic review protocol was published in PROSPERO on 20 March, 2025 (CRD420250624067), and amended on 29 July, 2025, to add more detailed methodology for gray literature.

### Inclusion and exclusion criteria

Inclusion criteria for peer-reviewed and gray literature are included in Table 1. Literature, including any United States K-12 school-based (population) F2S intervention or exposure involving the procurement and/or provision of local food from local food producers (intervention/exposure) and relationships with health outcomes (e.g., dietary intake and behavioral outcomes, laboratory values, weight, etc.) (outcomes) were eligible for inclusion. Health outcomes across multiple Nutrition Health Disparities Framework levels (child/student, family, producer, or community) were eligible for inclusion. Publication dates were limited to between 2002, when the USDA implemented the pilot Fresh Fruit and Vegetable Program, and the date of final article searches performed in 2025. Types of gray literature eligible for inclusion were proceeding papers, meeting abstracts, editorial material, conference papers and proceedings, dissertations and theses, reports, speeches and presentations, and working papers. Gray literature had to include adequate detail on study methods and results to be included. Literature reporting studies that were conducted outside the United States; that did not include K-12 schools or were not K-12-school–based; that did not have clear evidence of procurement and/or provision of local foods within F2S programs; that only reported outcomes like knowledge, preferences, or perceptions of food; and/or that were case studies, case series, or reviews were not eligible for inclusion.

**TABLE 1:** Inclusion and exclusion criteria used to screen peer-reviewed and gray literature.

	Include	Exclude
Population	− K-12 schools of any type (public, private, charter, and Tribal) in the United States − Program serves K-12 students in participating schools	− Non-K-12 schools (e.g., preschool/early childhood, university, and college) − Programs do not serve K-12 students in participating schools (e.g., community-serving) − Outside the United States
Intervention	− Any school-based F2S intervention/exposure involving the procurement and/or provision of local food from local food producers^1^	− Intervention/exposure not school-based − No procurement and/or provision of local food from local food producers included in F2S intervention (e.g., only school gardens, farm tours, etc.)
Comparators	− No comparator or − Comparison of F2S with standard procurement and/or provision of non-local food in K-12 schools	− —
Outcomes	− Child/student, family, producer, and community health outcomes [any outcome directly related or relevant to general health (e.g., dietary intake and behavioral outcomes, laboratory values, weight, quality of life, food security status, chronic disease status, health disparities, etc.)]^2^ − Must include results/outcomes	− Only outcomes not related to health (e.g., knowledge, preferences, perceptions of food, etc.) − Does not include results/outcomes or does not include results/outcomes described in sufficient detail
Timing	− Any study duration	− —
Study Design (for peer-reviewed literature)	− Primary observational (i.e., cross-sectional, case-control, and cohort) and experimental studies (i.e., randomized controlled trial, and quasi-experimental)	− Case studies and case series − Reviews (systematic, narrative, etc.)
Type (for gray literature)	− Proceeding papers, meeting abstracts, editorial materials, conference papers and proceedings, dissertations and theses, reports, speeches and presentations, and working papers	− Other
Publication date	− 2002–2025	− Prior to 2002
Language	− English	− Not English

Abbreviation: F2S, farm-to-school.

1 The definition of “local” could vary by school/program; anything that authors refer to as “local” was eligible for inclusion.

2 Although not originally included in inclusion criteria, health outcomes among teachers were reported by 1 study and included in data extraction and synthesis.

Among studies that met inclusion criteria, 3 secondary outcomes were also considered: economic impacts of local food procurement within F2S programs, barriers to local food procurement in F2Ss, and facilitators to local food procurement in F2S programs. Secondary outcomes were extracted from study results sections only, not from the study authors’ discussion or conclusions.

### Databases

In consultation with a research librarian, the following databases were searched for peer-reviewed literature: PubMed, Web of Science Core Collection, EconLit, Education Research Complete, and PAIS Index. Additionally, Web of Science Core Collection, ProQuest, GovInfo, and USDA Ag Data Commons were searched for gray literature.

### Search strategy

Search strategies for each database for peer-reviewed articles were developed in consultation with the research librarian. Pilot searches were conducted in December 2024 and January 2025. Search terms and results were reviewed by the full study team before finalizing search strategies. Final search strategies and results are available in Supplementary Appendix A. For all databases, a complex search was conducted combining terms, and a simple search was conducted, which included the term “farm-to-school” only, with filters for the United States and publication date. Peer-reviewed literature searches were conducted on 25 January, 2025 (PubMed, Education Research Complete, EconLit, and PAIS Index). Web of Science Core Collections was added as a database to ensure all relevant articles were retrieved and was searched on 5 March, 2025.

For gray literature, simplified searches were conducted, limited to the types of evidence listed above. Gray literature search strategies and a summary of results are included in Supplementary Appendix B. Gray literature searches were conducted on 1 May, 2025.

As a final potential source of relevant literature, reference lists for all peer-reviewed and gray literature included were reviewed. If any references were identified as potentially eligible for inclusion, a full-text review was conducted by one team member and confirmed by a second team member.

### Article selection process

For peer-reviewed articles, search results were uploaded to Covidence and deduplicated using Covidence’s automatic process before screening. For title and abstract screening, 2 team members (LL, KEC, DGP, and OMT) independently reviewed each record and indicated the decision to “include” or “exclude” using inclusion and exclusion criteria; the use of “maybe” was discouraged. If 1 or 2 reviewers selected “maybe,” the record was treated as a conflict. A third reviewer (LL, KEC, and MPP) resolved conflicts. The same process was used for full-text screening. Reasons for exclusion were recorded during full-text screening.

For gray literature, search results were saved as Excel files, and 1 reviewer (K.C.) performed title and abstract screening. A second reviewer (L.L.) completed full-text screening, ultimately deciding whether the source was eligible for inclusion according to the inclusion and exclusion criteria.

### Data extraction

During the full-text screening process, 2 team members developed a data extraction tool in Qualtrics (Supplementary Appendix C) and a codebook. The data extraction tool captured details related to study design, size, duration, location, objectives/aims, school and participant characteristics, F2S components and intensity of exposure, health outcomes, and Nutrition Health Disparities Framework level for health outcomes reported (individual, family/organization, community, and population) [8]. In addition, study findings related to economic impacts (perceived and measured) and local food procurement barriers and facilitators were captured. The data extraction tool was reviewed by all members of the study team, pilot tested with 1 peer-reviewed article, and finalized based on feedback and pilot test results. For the included peer-reviewed and gray literature, 2 team members extracted data for each source. Discrepancies were noted and discussed between the 2 team members until a consensus was reached.

### Risk of bias assessment

The Academy of Nutrition and Dietetics Quality Criteria Checklist (QCC) for primary research studies was used to assess the risk of bias of included peer-reviewed studies [20]. QCCs were completed by both data extractors. Any discrepancies were discussed and resolved between the 2 data extractors (K.C. and L.L.). Using the Academy’s criteria, articles were determined as positive, neutral, or negative according to risk of bias. Gray literature was not assessed for risk of bias.

### Data synthesis

Meta-analyses were not planned for this systematic review. Therefore, data synthesis is limited to narrative descriptions of included literature, presented by literature type (peer-reviewed compared with gray literature), with conclusions synthesized for health outcomes by level (child, family, producer, and community). Some studies had a broader set of goals or objectives, which are listed in narrative summaries below, but only results relevant to the objective of this systematic review are reported.

## Results

**FIGURE 1:**
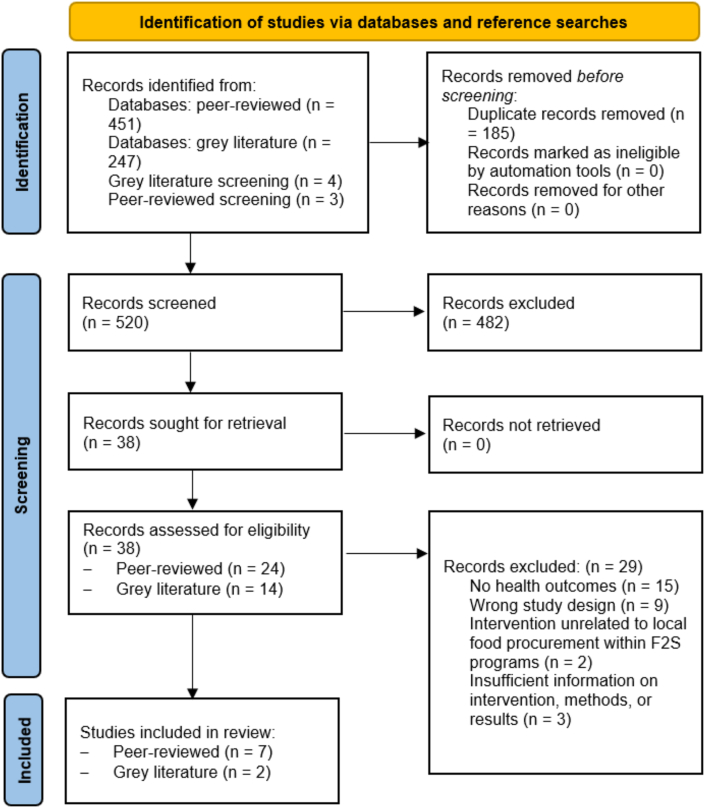
PRISMA flow diagram of the study selection process.

Figure 1 shows the inclusion and exclusion process for peer-reviewed and gray literature. Seven hundred 5 total records were retrieved from peer-reviewed database searches (n = 451), gray literature database searches (n = 247), and from gray literature (n = 4) and peer-reviewed source reference screening (n = 3). After 185 duplicates were removed, 520 records underwent title and abstract screening. Of these, 482 were excluded, leaving 38 records sought and retrieved for full-text screening. Twenty-nine records were excluded during full-text screening, primarily due to not including health outcomes (n = 15) or wrong study design (n = 9). Seven peer-reviewed articles and 2 gray literature sources met the inclusion criteria and are included in this review.

### Peer-reviewed literature summary

Of the 7 peer-reviewed articles included, 5 were quasi-experimental studies [[21], [22], [23], [24], [25], 1[21–25], 1 was a prospective cohort study [26], and 1 was a cross-sectional program analysis [27]. All studies collected quantitative data, and 3 collected both quantitative and qualitative data [24,26,27]. Forty elementary schools, 3 middle schools, and 3 school districts across 7 states (Alaska, California, Connecticut, Florida, Mississippi, South Carolina, and Wisconsin) were represented in studies, which included approximately 2914 students; Jones et al. [24] and Kropp et al. [25] present average student enrollment at F2S and control schools instead of individual-level student participation data. Approximately 269 parents/caregivers and 100 teachers and school administrators were represented, though the exact number of parents, teachers, and administrators that participated in focus groups was not presented in Jones et al. [24]. All studies included individual-level child health outcomes. Just 1 study presented individual-level health outcomes for other groups (teachers) [27]. This study also included limited information on parents’ perceptions of F2S impacts on families [27]. No studies examined producer or community health outcomes.

Local food procurement was the primary reported focus of the F2S program in just 2 studies [23,25]; all studies’ F2S programs included other activities (i.e., education, farm visits, cooking classes, and local food promotion) in addition to local food procurement. Studies are summarized below and in Table 2.

**TABLE 2:** Description of included peer-reviewed and gray literature evidence.

Author, year (type)	Study design and objective	Setting	Subjects	F2S program local food component	Outcomes and measures (NHDF level)	Results	Risk of bias
Barnard et al. [27], 2020 (peer-reviewed)	Cross-sectional program evaluation examining the implementation and impact of the Good Food for Oxford Schools program.	One public school district in Mississippi	239 parents/ caregivers of students in K-12 (*M* age = 40.7 y, 81% female, 78% White). 79 K-12 teachers, teaching assistants, and principals (*M* age = 40.9 y, 78% female, 89% White).	Students served at least 1 locally grown item per week; seasonal fruits and local food taste tests offered; scratch-cooked item offered daily >1 semester. Other F2S activities: school gardens, classroom lessons, student and family cooking events, afterschool programming.	Child-level F&V intake (home and school) and behaviors (trying new food and eating healthier food) assessed via parent/caregiver survey and teacher survey. Teacher-level F&V intake assessed via teacher survey. Parent perceptions of family-level program impacts assessed via parent/caregiver survey.	Parents agreed/strongly agreed the program: − Increased number of F&V child eats at school (56%) and home (46%) − Encouraged eating healthier foods (71%) − Encouraged trying new or different foods (66%) Teachers agreed/strongly agreed the program: − Increased number of F&V they eat (54%) − Helped them make healthier food choices (53%) Parent comments: program positively impacted children’s health (e.g., weight loss, eating health food) and family food choices.	Negative
Bersamin et al. [21], 2019 (peer-reviewed)	Quasi-experimental study (with comparison group) aiming to evaluate the preliminary efficacy of a school-based, food systems program, Neqa Elicarvigmun, on middle and high school students’ diet quality, traditional food intake, and attitudes and beliefs in remote Yup’ik communities.	Middle and high schools in 1 rural school district in 2 low-income communities in Alaska	76 middle and high school students (*M* age = 14.1 y, 55% female, 99% Yup’ik). 38 in F2S program community, 38 in comparison community.	Locally-caught salmon served in school lunches weekly >9-mo program. Other F2S activities: 5 culturally-responsive experiential classroom lessons, 4 intergenerational community events.	Child-level fish servings and diet quality (HEI) assessed via single 24-h recall and validated biomarker of fish and marine mammal intake; measurements at baseline, 4 mo, and 9 mo.	Among children in the F2S program and comparison group: − Any and total servings of fish increased Among children in the F2S program group: − Total fish servings, fish intake biomarker, diet quality increased more than comparison group	Neutral
Bontrager Yoder et al. [22], 2014 (peer-reviewed)	Quasi-experimental pretest-posttest evaluation to assess a F2S program’s effectiveness in improving students’ knowledge, attitudes, and behaviors relative to F&V consumption.	Nine elementary schools, primarily rural, in Wisconsin participating in AmeriCorps F2S.	1117 third to fifth graders (*M* age = 9.6 y, 47% female, 81% White).	Schools could select from 14 activities; 7/9 included farmers selling to school district, 8/9 included local items on lunch menus and 6/9 on breakfast menus >1 SY. Other F2S activities: farmer and farm visits, classroom and cafeteria tastings, classroom lessons and F&V snacks, fundraisers, information for parents, and school gardens.	Child-level fruit and vegetable intake (cups/1000 kcal and adequate intake based on USDA recommendations) assessed via 77-item FFQ (past 7 days) at baseline (start of SY) and follow-up (end of SY). Child-level F&V consumption (0%–100%) assessed via lunch tray photos.	Across 1 y: − No change in fruit or vegetable intake (cups/1000 kcal) among all children. − Fruit and vegetable intake decreased significantly among children with adequate intake at baseline. − Fruit intake increased significantly among children with low or very low intake at baseline. − % of children with adequate veg intake doubled from 4.3%–8.6% (*P* < 0.001). − % of children with adequate fruit intake unchanged (55.5%–55.1%); however significant change in distribution (*P* < 0.001). No difference in children’s F&V consumption per lunch tray photo analysis.	Neutral
Chiero et al. [23], 2021 (peer-reviewed)	Quasi-experimental study (with control group) aiming to determine impact of local F2S food procurement and messaging program on changes in elementary school students’ nutrition-related attitudes, self-efficacy, behaviors, and locally grown vegetable choices.	Six elementary schools in 1 urban school district in Connecticut	239 third to fifth graders (*M* age = 9.9 y, 49% female, 50% Hispanic, 85% FRPL-eligible). 81 in “Local Message” group, 79 in “Nutrition Message” group, and 79 in “Control” group	All 3 groups received local vegetables (kale, green beans, zucchini, butternut squash, and beets) served in cafeteria 10 times >16-wk program. Other F2S activities: “Local” and “Nutrition” groups received lunchroom marketing component and biweekly hands-on food-based classroom nutrition education.	Child-level nutrition-related behaviors assessed via 27-item survey. Child-level selection of local vegetables (yes, no) assessed via daily production records.	From pre- to postprogram implementation: − No difference within groups in children’s nutrition-related behaviors. − Children in local group’s overall nutrition behavior score increased vs. control.	Positive
Jones et al. [24], 2015 (peer-reviewed)	Quasi-experimental study (with control group) examining the impact of the first year of a F2S program on children’s consumption of fresh F&V and the perceptions of parents of effects on families’ food choices.	18 elementary and middle schools (12 intervention, 6 control) across 3 regions of South Carolina Parents from 5 schools participated in 5 focus groups (4–8 parents per focus group); 2 groups also included teachers or administrators	Student-level data not collected. Administrative data (average per school): enrollment = 528 students, FRPL participation = 62%	F2S program included partnering with local farmers, farmers’ markets, and food distributors to offer at least 2 F&V in the school cafeteria per month >1 SY. Other F2S activities: food promotion in cafeteria, nutrition and agriculture education in classrooms, F2S TA for school staff, field trips, and school gardens.	Child-level fruit and vegetable servings consumed assessed via photographic plate waste method (number of plates not reported).	In adjusted analyses, no significant difference in children’s vegetable or fruit intake between F2S and control schools. Focus groups themes did not include health impacts.	Neutral
Kropp et al. [25], 2018 (peer-reviewed)	Quasi-experimental study (with control group) examining effects of serving locally-procured produce as part of a F2S program on the selection and consumption of F&V served as part of the NSLP.	Six public elementary schools (3 F2S program, 3 control), primarily low-income, in 1 school system Florida	Student-level data not collected. Administration data (average per school): enrollment = 627 students, FRPL participation = 54%, 47% White and 31% Black	Local products, primarily raw vegetables, served in cafeteria on approximately 50 d during ∼4-mo study period. Other FTS activities: promotion of local products, 2 schools had gardens.	Child-level fruit and vegetable intake (average number of daily servings) assessed via 11,262 plate waste observations over 3-d periods at pre- and postprogram implementation.	Children’s fruit intake: − Decreased significantly from pre to post implementation in F2S program and control schools. Children’s vegetable intake: − Increased significantly from pre to post in F2S program and control schools. Adjusted OLS analyses: children at F2S program schools consumed significantly more servings of vegetables but not fruits after program implementation.	Neutral
Wang et al. [26], 2010 (peer-reviewed)	Prospective cohort evaluating effect of a comprehensive, multicomponent, school-based F2S program on nutrition-related outcomes, namely knowledge, attitudes, and behaviors.	Four public elementary schools in 1 mid-sized school district in California	327 fourth and fifth graders at baseline (58% female; 31% mixed, other, or unknown race) 18 teaching and administrative staff participated in key informant interviews	F2S program provided students with healthy, appealing, seasonal school meals made from locally grown and sustainable ingredients >1 SY. Other F2S activities: experiential learning in instructional gardens, cooking classes, connecting dining room to formal academic subjects.	Child-level fruit, vegetable, and total F&V intake in cups/day assessed via 3-d food diary (Tues-Thurs) in Y1 (2006–2007 SY) and Y2 (2007–2008 SY), compared between students attending schools with low, medium, or high F2S program implementation.	Significant increase in children’s vegetable and total F&V intake in schools with high F2S program implementation at baseline and Year 2. Significant decrease in children’s vegetable and total F&V intake in schools with low F2S program implementation at baseline and Year 2. No changes children’s in fruit intake regardless of F2S program implementation level. Interviews: no results reported	Neutral
Bobronnikov et al. [28], 2021 (gray)	Cross-sectional, national survey of SFAs’ participation in and perceptions of F2S programs.	Public, private, and charter SFAs in all 50 states as well as American Samoa, Guam, the Northern Mariana Islands, Puerto Rico, the United States Virgin Islands, and Washington DC that participated in the NSLP in SY 2018–2019	12,634 SFAs total (8393 SFAs participated F2S); 64% rural, 61% small (<1000 students)	SFAs select which of 30 distinct F2S activities they participated in during the 2018–2019 SY: 88% participated in at least 1 procurement activity. Of these, 66% also participated in promotion and/or education activities. Nearly half (46%) participated in ≥7 activities.	SFAs’ perception of child-level F2S benefits assessed through survey: which of the following benefits do you feel has resulted from your participation in F2S activities?	27% of F2S SFAs selected “increased consumption of items in school meals” among children. More very large F2S SFAs (48%) reported children’s increased consumption of items in school meals compared with small SFAs (22%; *P* < 0.01).	—
Machata et al. [5], 2024 (gray)	Cross-sectional, national survey of SFAs’ participation in and perceptions of F2S programs.	SFAs in all 50 states, the District of Columbia, Puerto Rico, Guam, the US Virgin Islands, the Northern Mariana Islands, and American Samoa that participated in the NSLP in SY 2022–2023	11,803 SFAs total (8,822 SFAs participated in F2S); % rural not provided, 68% small (<1000 students)	SFAs select which of 30 distinct F2S activities they participated in during the 2022–2023 SY: 63% served local food to students. Other F2S activities: agricultural education and edible school gardens (49%), promotion of local foods (45%).	SFA’s perception of child- level F2S outcomes assessed through survey: which of the following student/food service/community outcomes have resulted from your SFA’s participation in F2S activities?	SFAs perceived child-level outcomes: − Increase in consumption of F&V in school meals (61%) − Increased consumption of items other than F&V in school meals (29%) SFAs that participated in farm to school for ≥3 y reported higher rates of all student-related outcome measures including increased consumption of F&V in school meals than SFAs who participated in F2S for <3 y.	—

Abbreviations: F2S, farm-to-school; F&V. fruit and vegetable; FRPL, free and reduced-price lunch; HEI, Healthy Eating Index; NHDF, Nutrition Health Disparities Framework; NSLP, National School Lunch Program; OLS, ordinary least squares; SFA, school food authority; SY, school year.

### Gray literature summary

The 2 gray literature sources included in this review are the 2019 and 2023 USDA Farm to School Census reports [5,28]. Reports present nationally representative, quantitative, cross-sectional survey data collected from School Food Authorities across the United States and its territories. Results focus on School Food Authorities’ perceptions of child-level health outcomes, although food service and community outcomes (not related to health) were assessed in the 2023 Census. The 2019 report supplemented quantitative results with interviews with foodservice directors and producers, although thematic analysis was not conducted, so results are not included here [28].

### Health outcomes: F&V intake

Five peer-reviewed studies [22,[24], [25], [26], [27]] and 1 gray literature source [5] included fruit and/or vegetable intake or perceived intake as an outcome (Table 3). One peer-reviewed study [27] and the 2023 Farm to School Census Report [5] found at least 1 parent, teacher, or school administrator perceived improvement in child F&V intake. One peer-reviewed study found that teachers perceived an increase in their own F&V intake [27]. All 4 studies that measured children’s fruit intake through plate waste or dietary intake assessment found no significant change in adjusted analyses [22,[24], [25], [26]]. One study, however, also examined fruit intake by adequacy, defined using USDA food guide recommendations for mean age- and gender-specific energy requirements according to the Dietary Reference Intakes, and found improvements in adequate fruit intake, primarily among children with very low initial fruit intake [22]. Among 4 studies that measured children’s vegetable intake through plate waste or dietary intake assessment, 2 found improvements in vegetable intake [25,26], one found no change [24], and one found an improvement in vegetable adequacy according to USDA food guide recommendations (but no change in intake) [22] after adjustment for participant or school-level characteristics.

**TABLE 3:** Summary of articles included and direction of findings^1^ for health outcomes by Adapted Nutrition Health Disparities Framework Level.

Health outcome	Adapted Nutrition Health Disparity Framework Level				
	Individual			Family	Community
	Children	Teachers^2^	Producers		
F&V intake	Barnard et al. [27] 3 + 2023 F2S Census [5] 3 + Bontrager Yoder et al. [22] ∼ Wang et al. [26] +	Barnard et al. [27] 3 +	—	—	—
Fruit intake	Bontrager Yoder et al. [22] ∼ (intake) Bontrager Yoder et al. [22] + (adequacy) Jones et al. [24] ∼ Kropp et al. [25] ∼ Wang et al. [26] ∼	—	—	—	—
Vegetable intake	Bontrager Yoder et al. [22] ∼ (intake) Bontrager Yoder et al. [22] + (adequacy) Jones et al. [24] ∼ Kropp et al. [25] + Wang et al. [26] +	—	—	—	—
Intake of other foods (non-F&V)	Barnard et al. [27] 3 + (“health foods”) 2019 F2S Census [28] 3 + (“items in school meals”) Bersamin et al. [21] + (fish intake)	—	—	—	—
Diet quality	Bersamin et al. [21] +	—	—	—	—
Food behaviors	Barnard et al. [27] 3 + Chiero et al. [23] 3 +	Barnard et al. [27] 3 +	—	Barnard et al. [27] 3+	—
Weight loss	Barnard et al. [27] 3 +	—	—	—	—

NOTE. None means no articles included in this systematic review examined this health outcome in this group.

Abbreviations: F&V, fruit and vegetable; F2S, farm-to-school.

1 Increase/improvement +, no change ∼, or decrease in fully-adjusted models.

2 Emerged as a group during data extraction.

3 Perceived outcome

Barnard et al. [27] evaluated 239 parent and caregiver and 79 teacher, teaching assistant, and school principals’ perceptions of the Good Food for Oxford Schools program in one public school district in Mississippi. The program included the provision of at least 1 locally grown item per week in school cafeterias over the course of 1 semester. Parents who completed a program survey said that they agreed or strongly agreed that the program increased the number of F&V their child ate at school (56%) and at home (46%). Teachers who completed a program survey agreed or strongly agreed the program increased the number of F&V they ate themselves (54%).

Bontrager Yoder et al. [22] conducted a quasi-experimental pretest-posttest evaluation assessing associations between F2S program participation in 9 Wisconsin elementary schools and F&V knowledge, attitudes, behaviors, and intake among 1117 students over the course of 1 school year. Participating schools could select from 14 F2S activities; 7 of 9 schools purchased food from farmers, 8 of 9 included local items on lunch menus, and 6 of 9 included local items on breakfast menus. Children’s F&V intake was self-reported via the Block Kids 77-item food frequency questionnaire (FFQ). Across the school year, there was no change in mean total fruit intake [mean difference = −0.04 cups per 1000 kcal (SD = 0.85), *P* = 0.42] or vegetable intake [mean difference = −0.01 cups per 1000 kcal (SD = 0.49), *P* = 0.65] among all students. When students were stratified by years of prior F2S exposure (0, 1, and >2 y), those with >2 y had a significant decrease in mean fruit intake from baseline to follow-up [mean difference = −0.11 cups per 1000 kcals (SD = 0.06), *P* = 0.03] but there were no differences for vegetable intake, after adjusting for grade, percentage of students eligible for free/reduced-price lunch, and school.

Changes in fruit and vegetable intake were also examined by students’ baseline intake level (very low, low, or adequate) according to USDA food guide recommendations. Mean fruit intake decreased among children with adequate baseline fruit intake (≥2 servings per day) at follow-up [mean difference = −0.36 cups per 1000 kcal (SD = 0.88), *P* < 0.001] as did mean vegetable intake [mean difference = −1.00 cups per 1000 kcal (SD = 0.86), *P* = 0.001]. On the contrary, mean fruit intake increased among students with very low fruit intake [mean difference = 0.46 cups per 1000 kcals (SD = 0.65), *P* < 0.001] and low fruit intake [mean difference = 0.25 cups per 1000 kcals (SD = 0. 56), *P* = 0.001] at baseline; however, vegetable intake did not change significantly among those with very low or low vegetable intake at baseline. The distribution of fruit and vegetable adequacy levels changed significantly from baseline to follow-up for fruit intake and vegetable intake (*P* < 0.001). The percent of students with adequate vegetable intake increased from 4.3% (baseline) to 8.6% (follow-up). Changes remained significant when students were stratified by prior F2S exposure.

According to a separate observed plate waste analysis, there was no difference in the number of F&V cups that disappeared from students’ trays (assumed to be consumed) from baseline to the end of the school year [mean difference = −0.01 cups of F&V (SD = 0.37), *P* = 0.14], after adjusting for grade, percentage of students eligible for free/reduced-price lunch, and school. Differences were also not observed when students were stratified by years of prior F2S program exposure.

Jones et al. [24] conducted a quasi-experimental study evaluating changes in students’ F&V intake, estimated by photographic plate waste, during the first year of an F2S program in 12 elementary and middle schools across 3 regions of South Carolina compared with 6 control schools. The F2S program included partnering with local farmers, farmers’ markets, and food distributors to offer at least 2 F&V in the school cafeteria per month over the course of 1 school year. After controlling for matched pairs of F2S and control schools, there was no significant difference in vegetable intake (servings) between students attending F2S and control schools (β = 0.11, *P* > 0.05); however, students at F2S schools consumed significantly less fruit servings than students at control schools (β = −0.10, *P* < 0.05). The decrease in fruit intake was attenuated when researchers controlled for schools offering a la carte snacks during lunch.

Kropp et al. [25] conducted a quasi-experimental study examining the impact of serving locally-procured produce as a component of an F2S program. They evaluated changes in F&V selection and intake estimated by the quarter-waste method, a validated measure of plate waste. Six primarily low-income public elementary schools from 1 school district in Florida either implemented the F2S program (n = 3) or served as control schools (n = 3). Student-level data were not collected; however, based on administrative data, average enrollment was 627 students per school. Local products, primarily raw vegetables, were incorporated into school lunch menus and served in school cafeterias on ∼50 d during the 4-mo study period. Plate waste data were analyzed for 11,262 meal observations across the 6 schools to determine F&V selection and intake within and between the 3 F2S program and 3 control schools. At follow-up, students at F2S program schools had significantly higher consumption of fruits (0.466 vs. 0.300 servings, *P* <0.001) but not vegetables (0.215 vs. 0.202 servings, *P* = 0.32) compared with students at control schools. Within both F2S program and control schools, in unadjusted models from baseline to follow-up, students’ mean vegetable intake increased [F2S program = 0.167–0.215 servings (*P* < 0.001), control = 0.161–0.202 servings (*P* < 0.001)] but mean fruit intake decreased [F2S program = 0.493–0.466 servings (*P* = .007), control = 0.357–0.300 servings (*P* < .001)]. In models adjusted for school-level fixed effects, however, compared with students at control schools, students at F2S program schools consumed 0.107 more mean servings of vegetables (*P* = < 0.001) after F2S program implementation, representing a 65% increase over mean vegetable intake at baseline. Differences in fruit intake were not statistically significant (0.030 servings, *P* = 0.29).

Wang et al. [26] conducted a prospective cohort study evaluating changes in nutrition-related outcomes, namely knowledge, attitudes, and behaviors, including F&V intake, after implementation of a comprehensive, multicomponent, school-based F2S program in 4 public elementary schools in 1 mid-sized school district in California [26]. Fourth and fifth-grade students (n = 327) were provided with healthy, appealing, seasonal school meals made from locally grown and sustainable ingredients over the course of 2 school years. For analyses, students were categorized based on the degree of F2S program implementation at their school (high, medium, or low), and F&V intake was measured using 3-day (Tuesday through Thursday) food diaries. Students attending schools with higher F2S program implementation at baseline and year 2 had significant increases in mean daily vegetable intake (0.83–1.30 cups, *P* < 0.05) and total mean daily F&V intake (2.15–2.64 cups, *P* < 0.05), after adjusting for grade, race/ethnicity, parent/guardian education, and baseline consumption. Students attending schools with low F2S program implementation at baseline and year 2 had significant decreases in total mean daily F&V intake (2.23–1.91 cups, *P* < 0.05), after adjusting for the same covariates. Regardless of the F2S program implementation level, mean fruit intake did not change significantly from baseline to year 2.

The 2023 Farm to School Census Report included responses from 11,803 School Food Authorities, of which 8822 (75%) participated in at least 1 F2S activity in the 2022 to 2023 school year [5]. Rurality of participating School Food Authorities was not reported, but 68% were classified as small, serving <1000 students. In the 2023 Farm to School Census, 61% of School Food Authorities participating in F2S selected “increase in consumption of F&V in school meals” as a F2S outcome [5]. More School Food Authorities participating in F2S for ≥3 y reported this increase (67%) compared with School Food Authorities participating in F2S for <3 y (56%), although tests of statistical significance were not reported.

### Health outcomes: intake of other foods

Two peer-reviewed studies assessed consumption of “other foods” (non-F&V) [21,27], and the 2019 Farm to School Census assessed students’ overall consumption of “items in school meals” [28].

Barnard et al. [27] found parents perceived that the F2S program was positively associated with children’s consumption of “health foods” at home.

Bersamin et al.’s [21] quasi-experimental study evaluated changes in middle and high school students’ salmon intake after implementation of a school-based, 9-mo food systems program. This was the only study found during this review that focused on procuring and serving fish or animal-based products in an F2S program. Middle and high school students (n = 76) from 1 rural school district in 2 low-income communities in Alaska participated. Locally-caught salmon was served in school lunches weekly over the 9-mo program. Fish intake was assessed via a single 24-h recall collected using a multiple-pass approach at baseline, 4 mo, and 9 mo. In both the F2S program and comparison group, any total servings of fish increased during the study period. The F2S program group had a significantly higher likelihood of any fish servings [aOR = 1.85 (95% CI = 1.00, 3.45)] and total fish servings [aRR = 2.22 (95% CI = 1.12, 4.35)] compared with the comparison group, after adjusting for age, sex, and way of life (traditional vs. White); however, the F2S program × time interaction term was not significant for any or total fish servings. A biomarker of fish intake was also assessed and increased more in the F2S program group compared with the comparison group [F2S program condition × time β = 0.16 (SE = 0.08), *P* = 0.05], controlling for the same covariates.

The 2019 Farm to School Census report included responses from 12,634 School Food Authorities, of which 8393 (66%) participated in at least 1 F2S activity in the 2018 to 2019 school year [28]. Sixty-four percent of School Food Authorities were rural, and 61% were classified as small, serving <1000 students. In the 2019 Farm to School Census, 27% of School Food Authorities participating in F2S selected “increased consumption of items in school meals” as a F2S program benefit. Significantly more very large School Food Authorities reported increases in school meal item consumption compared with smaller School Food Authorities (48% vs. 22%, *P <* 0.01).

### Health outcomes: diet quality

One peer-reviewed study included diet quality as an outcome [21]. In addition to measuring fish intake, Bersamin et al. [21] also evaluated changes in middle and high school students’ diet quality after implementation of the school-based, nine-month F2S food systems program. The authors used Healthy Eating Index (HEI)-2010 scores as a proxy for diet quality. HEI scores were calculated from Nutrition Data System for Research output files obtained through a single 24-h recall collected using a multiple-pass approach at baseline, 4 mo, and 9 mo [21]. Diet quality increased more in the F2S program group than the comparison group over the 9-mo study period (F2S program condition × time β = 4.57 (SE = 1.99), *P* < 0.05), after controlling for age, sex, and way of life. The authors noted the F2S program was associated with improvements in overall diet quality among F2S program participants at the 4- and 9-mo time points, whereas diet quality declined among control participants at both follow-up time points.

### Health outcomes: other food behaviors

Two peer-reviewed studies included food-related behaviors other than dietary intake as outcomes, finding that parents and teachers perceived F2S programs with local food procurement to be associated with positive changes in student and teacher-level food choices, students’ vegetable-related nutrition behavior score, and family-level food choices [23,27].

Barnard et al.’s [27] survey of parents and caregivers found most agreed or strongly agreed the F2S program encouraged their child to eat healthier foods (71%) and encouraged their child to try new or different foods (66%) [27]. Parents further reported F2S program exposure was associated with positive changes in family-level food choices, illustrated by a single quote from an open-ended question. In this study, 53% of teachers also agreed or strongly agreed the program helped them make healthier food choices.

Chiero and Mobley [23] conducted a quasi-experimental study examining self-reported changes in elementary school students’ nutrition-related attitudes, self-efficacy, behaviors, and locally grown vegetable choices after implementation of a 16-wk local F2S food procurement and messaging program. Outcomes of interest were assessed through a 27-item self-reported survey pre- and postprogram [23]. Six elementary schools in one urban school district in Connecticut were enroled, and 239 third through fifth-grade students participated. All 3 study groups [“local messaging” (n = 81), “nutrition messaging” (n = 79), and “control” (n = 79)] received 5 locally grown vegetables (kale, green beans, zucchini, butternut squash, and beets) served in the cafeteria 10 times over the 16-wk program. The local messaging group also received messages highlighting locally grown vegetables, whereas the nutrition messaging group received general nutrition messages related to USDA MyPlate. The local messaging and nutrition messaging groups also received biweekly hands-on nutrition education in the classroom. Postprogram, there was no difference among the 3 study groups in overall vegetable-related nutrition behavior score, which reflects knowledge, attitudes, self-efficacy, and preference for vegetables; however, a difference was detected among the 3 groups (*P* = 0.001) after adjustment for pre-program scores. According to post hoc analyses, the overall vegetable behavior score was significantly higher in the “local message” group (mean = 56.1 ± 11.2) than the nutrition message group (mean = 46.5 ± 13.2, no *P* value provided) and the control group (mean = 46.4 ± 96, *P* = 0.002).

### Other health outcomes

Just one study included any “other” health outcome. Barnard et al. [27] found parents perceived that the F2S program was positively associated with children’s health, specifically mentioning weight loss. These results were based on parents’ responses to open-ended survey questions only.

### Economic outcomes

One peer-reviewed study and both Farm to School Census Reports provided some information on perceived economic impacts of F2S programs, summarized here [5,24,28].

Jones et al. [24] conducted 5 focus groups with parents of elementary and middle school students. Parents mentioned that F2S benefits local farmers by providing income and noted they preferred to “give” their money to independent farmers and local food producers versus a store.

In the 2019 Farm to School Census, 17% of School Food Authorities selected “increased participation in school meals,” and 15% selected “lower school meal program costs” and “reduced food waste” as F2S benefits [28]. School Food Authorities also mentioned “benefits to the economy” as an “Other” F2S benefit. In the 2023 Farm to School Census, 41% of School Food Authorities reported “increased participation in school meals,” 33% reported “increased support for local businesses,” and 17% reported “lower school meal program costs” as F2S program outcomes [5]. School Food Authorities that purchased local food spent almost $1.80 billion on local food in school year 2022 to 2023, an increase from school year 2018 to 2019 ($1.26 billion); however, School Food Authorities spent a higher percent of overall food expenditures on local food purchasing (20%) in 2018 to 2019 compared with 2022 to 2023 (16%).

### Farm-to-school facilitators

Five peer-reviewed studies and the 2023 Farm to School Census report describe facilitators to local food procurement in F2S programs.

Barnard et al. [27] found that expanding local foods, requiring teachers to eat in the cafeteria, increasing exposure to new foods, including more visits to the cafeteria for taste testing, expanding program elements (i.e., enhancing middle school programming, cooking demos, and offering snacks), general support for the program staff, and connecting F2S to math and science standards could support implementation of F2S and local food procurement in F2S programs. Bersamin et al. [21] reported that the use of traditional foods and a cultural connectedness focus of the F2S program facilitated program success. Helping students develop skills to harvest and store salmon was specifically noted. Bontrager Yoder et al. [22] found that schools with more prior F2S programming experienced additional increases in students’ F&V variety and a lower percentage of students’ trays with no F&V. Jones et al. [24] reported charismatic program champions, parent engagement in programs, expanded program reach (i.e., afterschool programs), and pre-slicing foods such as fruit to enhance accessibility led to program success. Further, authors noted that district-level factors, such as implementation of multiple programs (e.g., F2S and the Fresh Fruit and Vegetable Program), teachers sitting with students while eating, and teacher involvement with school administration to create healthy environments, could further improve program implementation. Last, Wang et al.’s [26] results showed that higher degrees of F2S program implementation, reflecting more F2S program attributes (freshly prepared meals, food that is visually and aromatically appealing, tables and chairs available for eating, eating area free of commercial messaging, paid compared with volunteer garden specialist and cooking specialist teachers, hours of garden instruction and cooking class instruction per year, and lesson integration), led to more significant increases in students’ F&V intake.

The 2023 Farm to School Census asked schools about strategies that would encourage F2S participation [5]. Currently participating schools and schools that had previously participated but were not currently participating in F2S noted additional funding for the School Food Authority in general (51% and 35%), targeted funding for local food procurement (38% and 24%), and training/technical assistance on finding local foods (32% and 22%) as the top 3 strategies. School Food Authorities that were not currently participating in F2S but had plans to in the future reported similar needs but reported more need for training and technical assistance related to not only finding local food (42%), but also procurement and delivery processes (36%), paying for local foods (33%), and preparing meals with local foods (27%). Consistent with reported needs, School Food Authorities with greater kitchen capacity were more likely to participate in F2S, and 76% of School Food Authorities participating in F2S had at least 1 staff member with some portion of their time dedicated to F2S activities. Finally, 42% of School Food Authorities participating in F2S had wellness policies that support F2S, an increase from the 2019 Farm to School Census.

The 2023 census was the first to evaluate values-based procurement practices among School Food Authorities. Some School Food Authorities currently purchased food with the farm identity preserved (15%), which was certified USDA organic (10%), from woman-owned (10%) or minority-owned (8%) businesses, or with environmental certifications or indications (i.e., low-spray, integrated pest management, and organic transition) (5%). Many more School Food Authorities, however, indicated wanting to purchase foods with these characteristics in the future [minority-owned business (64%), woman-owned business (63%), environmental certifications or other indications (57%)].

### F2S barriers

Peer-reviewed studies did not report barriers to local food procurement in F2S program specifically; barriers presented were related to general F2S program participation and perceptions. Both Farm to School Census Reports included barriers to local food procurement in F2S programs as perceived by School Food Authorities.

The 2019 Farm to School Census found School Food Authorities experienced significant barriers to local food purchasing [28]. Sixty percent of School Food Authorities participating in F2S reported challenges in food availability while other noted procurement and delivery (51%), cost (37%), and staffing and school kitchen challenges (36%). Among specific challenges, limited availability of local foods was the most common (45%), followed by difficulty finding local producers, suppliers, and distributors (27%) and lack of local foods offered by primary vendors (26%). The 2019 Census also found School Food Authorities that were larger and had participated in F2S longer experienced more local food procurement challenges.

The 2023 Farm to School Census found that limited availability of local foods was the most common challenge experienced by School Food Authorities participating in F2S (42%), followed by cost (35%), procurement and delivery challenges (29%), and lack of staff/kitchen (18%) [5]. From 2018–2019 to 2022–2023, the percent of School Food Authorities reporting availability (31%–42%) and cost (19%–35%) as challenges increased notably. Among schools that previously participated in F2S, 42% selected lack of staff available to lead or conduct F2S activities, 22% selected unable to find vendors that provide local foods, and 19% selected lack of funds to conduct F2S activities as the top reasons for no longer participating.

### Study quality

Of the 7 peer-reviewed studies included in this review, 1 was rated positive [23, 5[23], 5 were rated neutral [21,22,[24], [25], [26]], and 1 was rated negative [27], suggesting the current literature base on local food procurement in F2S programs has a moderate-to-high risk of bias. Future studies would benefit from more rigorous study designs, when possible, such as including control groups, randomizing participants or schools, providing detailed descriptions of program components and assessments of exposure, and incorporating stronger measurements of various health outcomes at the individual, family, community, and population levels, such as weight status, indicators of chronic disease (i.e., blood pressure, cholesterol, and hemoglobin A1c), mental health, and health-related quality of life.

## Discussion

This systematic review included 7 peer-reviewed studies and 2 gray literature sources documenting relationships between implementation of local food procurement in United States F2S programs and child, teacher, and family health outcomes. Most F2S programs provided local produce and just 1 study included local sources of animal-based products (salmon) [21]. All studies focused on individual-level health outcomes among children, primarily children’s F&V intake. Just 1 study included self-reported, individual-level health outcomes among teachers and just 1 study included a single quote about family-level health outcomes [27]. We did not identify any studies that assessed relationships between local food procurement in United States F2S programs and producer or community-level health outcomes.

Overall, studies found that implementation of F2S programs with local food procurement was associated with increases in children’s vegetable intake or adequacy but may not change fruit intake. Longer participation in F2S (≥2–3 y) and exposure to multiple F2S activities in addition to local food procurement may be important to program success. Children with low or inadequate F&V intake may experience the most benefit from local food procurement in F2S programs. Interestingly, one study found that children with adequate F&V intake prior to F2S program implementation had decreases in intake [22]. Since diet quality is generally lower among older children compared with young children, F2S programs with local food procurement may be particularly important in middle and high schools [[29], [30], [31]]. In this review, only 3 peer-reviewed studies included middle and/or high schools [21,24,27]. Further, establishing positive dietary habits such as F&V consumption during the elementary school years may improve F&V preference, acceptability, and intake over time [32,33]. Studies carefully evaluating relationships between timing and dose of F2S program exposure and student outcomes could best examine whether F2S programs can mitigate the typically observed declines in diet quality. Most peer-reviewed studies in this review were short-term, with only Bontrager Yoder et al. [22] and Wang et al. [26] examining outcomes associated with ≥1 y of F2S program exposure. These studies observed that school-level factors, including higher intensity of F2S program implementation and more years of implementation, were positively associated with student-level increases in F&V indicators.

Combining local food procurement with other F2S activities such as local food promotion (i.e., messaging and signage), taste testing, and other activities may be advantageous. For example, Chiero and Mobley [23] found that the combination of local vegetable procurement and local vegetable messaging in the lunchroom was associated with improved vegetable-related behaviors. Local food marketing included youth-friendly messages, vibrant colors, and graphics that were tested with students prior to use.

It is important to note that F&V intake was not always measured using gold standard dietary assessment methodologies in studies included in this review. Just one study included a biomarker of dietary intake [21]. Future F2S studies should use strong, standardized measures of dietary intake and diet quality to assess F&V intake and other health outcomes. Conclusions could not be drawn for other child, teacher, or family health outcomes assessed in studies included in this systematic review (fish and other food intake, food-related behaviors, diet quality, and weight) due to limited evidence.

### Barriers and facilitators

Among included studies, barriers and facilitators to local food procurement in F2S programs were extracted and examined. The 2019 and 2023 Farm to School Census reports show schools overwhelmingly cite lack of staff time, funding, and/or access to local foods as barriers, consistent with the extant F2S literature [17,[34], [35], [36]]. Rising food costs and funding cuts also contribute significantly to local food procurement barriers in F2S programs. Although conducting this review, for example, the Patrick Leahy Farm to School Grant Program was cut and then reinstated [37]. Federal funding cuts and uncertainty cannot be controlled by schools and F2S program advocates, however, addressing other barriers (e.g., providing training and technical assistance to School Food Authority staff on finding, paying for, and transporting local food items; allocating dedicated staff time to F2S activities when funding permits; building partnerships and trust with local producers and food system; and incorporating F2S into school wellness policies) may encourage new and continued participation [[38], [39], [40]]. Of note, peer-reviewed studies included in this review did not focus on barriers to local food procurement in F2S programs.

F2S facilitators, on the other hand, provide opportunities to increase local food procurement and strengthen F2S implementation and participation, which may in turn result in larger changes in outcomes of interest. Schools can engage F2S program champions; limit competing a la carte or dessert items; and include multi-level, culturally-relevant F2S activities such as family and community engagement, field trips, local food marketing messages in cafeterias, and complementary classroom education. Further, directly purchasing from local producers may lower costs and improve order flexibility, reducing some administrative burden [41,42]. In addition to facilitators found in this review, boosting schools’ capacity and social capital (i.e., existing networks and relationships necessary to implement F2S), motivation to provide fresh and high-quality food that students prefer, and desire to contribute to the local economy and community value could enhance the implementation and sustainability of F2S programs [[41], [42], [43]]. Local, state, and federal policies and programs that provide F2S funding, resources, and technical assistance, such as the Patrick Leahy Farm to School Grant Program, may also bolster participation [44]. The alignment of local food procurement in F2S programs with schools’ increasing preference for values-based procurement practices, noted in the 2023 Farm to School Census, is also worthy of additional research.

Importantly, food producers’ perceptions of barriers and facilitators to local food procurement were not reported by any study included in this systematic review, and economic impacts were only reported from the perspective of parents and School Food Authorities versus formal economic analyses [45]. Local food producer outcomes must also be included in future F2S studies, and especially in research focused on local food and values-based procurement, since F2S may support positive local economic and producer health outcomes [46,47].

### Limitations

Limitations of this systematic review should be acknowledged. We were not able to conduct a meta-analysis because of variations in study design, intervention components, and study outcomes and the moderate-to-high risk of bias among the included studies. For peer-reviewed articles, title and abstract screening was performed according to full inclusion and exclusion criteria, which could have led to the exclusion of articles that did not specifically mention local food procurement or health outcomes in abstracts. We also believe a limited number of studies were excluded during full-text screening for not explicitly stating the F2S program included local food procurement. When unclear, study authors were contacted to confirm, but they did not always respond. A standard definition of “local” was not required for studies to be included and varied among the literature included.

In addition, all evidence in this systematic review evaluated F2S programs that included other F2S activities in addition to local food procurement. All the included studies were quasi-experimental or observational, and only 1 study used analytic methods designed to facilitate causal inference from observational data; this limits the ability to draw causal conclusions from the studies in this review. Moreover, studies were limited to publication between 2002 and 2025, potentially excluding sources of evidence published prior to the implementation of the Fresh Fruit and Vegetable Program in 2002. Gray literature searches were limited to 4 databases and did not include conference posters. Manual searches of websites for gray literature were not performed and title and abstract screening and full-text screening for gray literature was performed by only 1 reviewer at each step due to project time constraints.

Finally, we extracted secondary outcomes (economic impacts, barriers, and facilitators of local food procurement in F2S programs) from studies that met full inclusion and exclusion criteria only. Literature searches were designed to capture health outcomes, the review’s primary outcome, not secondary outcomes. Economic outcomes, barriers, and facilitators of local food procurement in F2S reported here are therefore not representative of the entire F2S literature, but our preliminary findings warrant additional investigation.

In conclusion, local food procurement as a component of F2S programming is associated with improvements in vegetable intake among K-12 children, especially for those with inadequate intake prior to program engagement, but not fruit intake. Additionally, F&V intake and variety may improve among students when they are exposed to multiple F2S program activities ≥2–3 y. These findings support the provision of federal and state funding for schools to implement and sustain F2S programs that include local food procurement as a strategy to improve child vegetable intake.

The current evidence is too limited to draw conclusions for any other health outcome included in this systematic review for children, teachers, families, producers, or communities. In the future, more rigorous assessment of multi-level health outcomes should be investigated in studies evaluating F2S programs. In the meantime, facilitators identified in this review, such as program champions, dedicated staff time, and parent and community involvement, and barriers to local food procurement must be addressed in partnership with schools and school districts, local food producers, and communities to maximize F2S program engagement.

## Author contributions

The authors’ contributions were as follows – KEC, LL, JDE: designed research; KEC and LL: conducted research and wrote manuscript; KEC, LL, DGP, OMT, EYJ, MPP: analyzed data; and all authors: provided content and suggestions for data extraction, revision, and have read and approved the final manuscript; LL had primary responsibility for final content.

## Data availability

Data described in the manuscript, code book, and analytic code will be made available upon request pending application and approval.

## Funding

This paper was supported by Healthy Eating Research.

## Conflicts of interest

There are no conflicts to report.
