# Supplementary material for: Multi-Level Health Outcomes of Local Food Procurement in United States Farm-to-School Programs: a Systematic Review
Source: Adv Nutr. 2025 Dec 31;17(2):100580. doi: 10.1016/j.advnut.2025.100580 (PMC12857378; doi:10.1016/j.advnut.2025.100580)
Supplement: multimedia component 1 [file mmc1.docx]

Appendix A: Summary of Peer-Reviewed Article Searches

Date of Searches: January 25, 2025

Date of Web of Science Search: March 5, 2025

Summary of Searches:

| Databases | Full Search | F2S Search | Total |
| --- | --- | --- | --- |
| PubMed | 57 | 51 | 108 |
| Education Research Complete | 12 | 50 | 62 |
| EconLit | 11 | 18 | 29 |
| PAIS Index | 19 | 39 | 58 |
| Web of Science* | 61 | 133 | 194 |
| Total Records Retrieved |  |  | 451 |
| Duplicates Removed |  |  | 183 |
| Total Records Available to Screen |  |  | 268 |

*Searched March 5, 2025

Table 1. Search Terms and Details

| Database | Strategy | Results |
| --- | --- | --- |
| PubMed Full Search | Search 1: ("Schools"[MeSH Terms] OR "primary school"[All Fields] OR "secondary school"[All Fields] OR "elementary school"[All Fields] OR "middle school"[All Fields] OR "high school"[All Fields] OR “school district”[All Fields] OR "K-12"[All Fields]) NOT "Universities"[MeSH Terms] | 177,649 |
|  | Search 2: "farm-to-school"[All Fields] OR "farm-to-school"[All Fields] OR "farm to institution"[All Fields] OR "local food"[All Fields] OR "seasonal food"[All Fields] OR "locally sourced"[All Fields] OR "local food procurement"[All Fields] OR "local food purchasing"[All Fields] | 1,985 |
|  | Search 3: “United States"[MeSH Terms] OR "United States"[All Fields] | 5,129,681 |
|  | Search 1 AND Search 2 AND Search 3 | 57 |
|  | Date filter: January 1, 2002 through January 31, 2025 | 57 |
|  | Total records retrieved | 57 |
| PubMed Confirmatory Search | Search 1: "farm to school"[All Fields] | 66 |
|  | Search 2: “United States"[MeSH Terms] OR "United States"[All Fields] | 5,129,681 |
|  | Search 1 AND Search 2 | 51 |
|  | Date filter: January 1, 2002 through January 31, 2025 | 51 |
|  | Total records retrieved | 51 |
| Education Research Complete Full Search | Search 1: DE "CHARTER schools" OR DE "ELEMENTARY schools" OR DE "PRIMARY schools" OR DE "PRIVATE schools" OR DE "PUBLIC schools" OR DE "RURAL schools" OR DE "SCHOOL districts" OR DE "SECONDARY schools" OR DE "URBAN schools" | 95,267 |
|  | Search 2: "farm to school" OR "farm to institution" OR "local food" OR "seasonal food" OR "locally sourced" OR "local food procurement" OR "local food purchasing" | 882 |
|  | Search 1 AND Search 2 | 42 |
|  | Geography filter: United States | 13 |
|  | Date filter: January 1, 2002 through January 31, 2025 | 12 |
|  | Total records retrieved | 12 |
| Education Research Complete Confirmatory Search | “farm to school” | 246 |
|  | Geography filter: United States | 59 |
|  | Date filter: January 1, 2002 through January 31, 2025 | 51 |
|  | Total records retrieved* | 50 |
| EconLit Full Search | Search 1: elementary school OR primary school OR secondary school OR middle school OR high school OR school district OR K-12 | 27,620 |
|  | Search 2: "farm to school" OR "farm to institution" OR "local food" OR "seasonal food" OR "locally sourced" OR "local food procurement" OR "local food purchasing" | 595 |
|  | Search 1 AND Search 2 | 26 |
|  | Geographic region filter: Northern America | 11 |
|  | Date filter: January 1, 2002 through January 31, 2025 | 11 |
|  | Total records retrieved | 11 |
| EconLit Confirmatory Search | “farm to school” | 21 |
|  | Geographic region filter: Northern America | 18 |
|  | Date filter: January 1, 2002 through January 31, 2025 | 18 |
|  | Total records retrieved* | 18 |
| PAIS Index Full Search | Search 1: MAINSUBJECT.EXACT("School environment") OR MAINSUBJECT.EXACT("Elementary schools") OR MAINSUBJECT.EXACT("Secondary schools") OR MAINSUBJECT.EXACT("School districts") OR MAINSUBJECT.EXACT("Public schools") | 9,333 |
|  | Search 2: “farm to school" OR "farm to institution" OR "local food" OR "seasonal food" OR "locally sourced" OR "local food procurement" OR "local food purchasing" | 2,030 |
|  | Search 1 and Search 2 | 48 |
|  | Location filter: United States | 20 |
|  | Date filter: January 1, 2002 through January 31, 2025 | 19 |
|  | Total records retrieved* | 19 |
| PAIS Index Confirmatory Search | “farm to school” | 99 |
|  | Location filter: United States | 39 |
|  | Date filter: January 1, 2002 through January 31, 2025 | 39 |
|  | Total records retrieved* | 39 |
|  | *One record could not be retrieved and was therefore excluded |  |
| Web of Science Full Search | Search 1: "elementary school" OR "primary school" OR "secondary school" OR "middle school" OR "high school" OR "school district" OR K-12 | 260,445 |
|  | Search 2: "farm to school" OR "farm to institution" OR "local food" OR "seasonal food" OR "locally sourced" OR "local food procurement" OR "local food purchasing" | 7,938 |
|  | Search 1 AND Search 2 | 95 |
|  | Location: USA | 63 |
|  | Date filter: 2002 through 2025 | 61 |
|  | Total records retrieved | 61 |
| Web of Science Confirmatory Search | “farm to school” | 157 |
|  | Location: USA | 133 |
|  | Date filter: 2002 through 2025 | 133 |
|  | Total records retrieved | 133 |

Appendix B: Summary of Grey Literature Searches

Date of Searches: May 1, 2025

| Database | Literature Types | Results |
| --- | --- | --- |
| Web of Science Core Collection | Proceeding Paper, Meeting Abstract, Editorial Material | 46 |
| ProQuest | Conference Papers & Proceedings, Dissertations & Theses, Reports, Speeches & Presentations, Working Papers | 109 |
| GovInfo | Other | 75 |
| USDA Ag Data Commons | No Specifications (all results are reports/data) | 17 |
|  | Total records retrieved | 247 |

Appendix C: Data Extraction Tool

Q1 Reviewer (your) name:

________________________________________________________________

Q2 Study title

________________________________________________________________

Q3 Type of literature (grey vs peer reviewed)

- Grey literature
- Peer reviewed literature

Q4 Year of publication

________________________________________________________________

Q5 DOI/link

________________________________________________________________

Q6 Study type

- Quantitative
- Qualitative
- Quantitative and qualitative
- Mixed methods
- Other (specify): __________________________________________________

Q7 Study design (please refer to the Academy's "Glossary of Terms Related to Research Design")

- Cross-sectional
- Case control
- Cohort
- Quasi-experimental
- Randomized controlled trial (RCT)
- Other (specify): __________________________________________________

Q8 Study duration (length of data collection)

________________________________________________________________

Q9 Study location (state) (if it is a multi-state study, list all)

________________________________________________________________

Q10 Inclusion criteria (at school/school district and student levels, if applicable)

________________________________________________________________

Q11 Exclusion criteria (at school/school district and student levels, if applicable)

________________________________________________________________

Q12 Study objectives/aims

________________________________________________________________

Q13 Farm-to-school related objective (if different than overall study objective)

________________________________________________________________

Q14 School type [e.g., public/private/charter, rural/urban - include detailed description if available (e.g., % public, % rural)]

________________________________________________________________

Q15 School level - select all that apply

1. Elementary school (K-5)
2. Middle school (grades 6-8)
3. High school (grades 9-12)
4. Not provided in the manuscript

Q16 Sample size: total # of schools/school districts

________________________________________________________________

Q17 Sample size: total # of students

________________________________________________________________

Q18 Participants' age (e.g., range, mean and SD, median and IQR, if provided)

________________________________________________________________

Q19 Sex distribution of participants

1. Boys (n, %) __________________________________________________
2. Girls (n, %) __________________________________________________
3. Non-binary (n, %) __________________________________________________
4. Not provided in the manuscript

Q20 Race/ethnicity distribution of participants

1. Hispanic (n,%) __________________________________________________
2. Non-Hispanic White (n, %) __________________________________________________
3. Non-Hispanic Black (n,%) __________________________________________________
4. Non-Hispanic Asian (n,%) __________________________________________________
5. Other Race - Including Multi-Racial (n, %) __________________________________________________
6. Not provided in the manuscript

Q21 Other participant characteristics if provided (e.g., rural/urban distribution, % free/reduced price)

________________________________________________________________

Q22 Farm-to-school intervention type(s) and description. Select all that apply.

1. Local food procurement
2. School gardens
3. Food, nutrition, and/or agriculture education
4. Farm field trips and on-farm experiences
5. Taste testing
6. Cooking and food preparation activities
7. Community and family engagement (Involves parents, caregivers, and community members)
8. Farmers Markets and Mobile Markets at Schools
9. Other (describe): __________________________________________________

Q23 Number of participants in F2S intervention group

________________________________________________________________

Q24 Farm-to-school intervention duration

________________________________________________________________

Q25 Farm-to-school intervention frequency

________________________________________________________________

Q26 Is there a comparator group in the study

- Yes
- No

Q27 Description of comparison group

________________________________________________________________

Q28 Number of participants in comparator group

________________________________________________________________

Q29 Health outcome(s): definition and measurement details (units should be included)

1. Dietary intake __________________________________________________
2. Behavioral outcomes __________________________________________________
3. Laboratory values __________________________________________________
4. Weight outcomes __________________________________________________
5. Food security status __________________________________________________
6. Chronic disease status __________________________________________________
7. Quality of life __________________________________________________
8. Health disparities __________________________________________________
9. Other (specify) __________________________________________________

Q30 Health outcomes: quantitative results

________________________________________________________________

Q31 Health outcomes: qualitative/thematic results

________________________________________________________________

Q32 Description of economic impacts/outcomes

________________________________________________________________

Q33 Description of farm-to-school barriers identified

________________________________________________________________

Q34 Description of farm-to-school facilitators identified

________________________________________________________________

Q35 Nutrition Health Disparities Framework (NHDF) level(s) of health outcomes - select all that apply NHDF available here.

1. Individual
2. Interpersonal (family/organizational)
3. Community
4. Society/population health

Q36 Nutrition Health Disparities Framework (NHDF) domain(s) of influence included in intervention - select all that apply

1. Biological
2. Behavioral
3. Physical/built environment
4. Sociocultural environment
5. Health care system

Q37 Description of mechanisms proposed by authors linking farm-to-school intervention to health outcome(s)

________________________________________________________________

Q38 Limitations identified by authors

________________________________________________________________

Q39 Authors' conclusions

________________________________________________________________

Q40 Author-identified research gaps and/or future directions

________________________________________________________________

Q41 Funding source

________________________________________________________________

Q42 Completed Quality Criteria Checklist (QCC), available here.

Q43 Quality assessment/risk of bias (Academy Quality Criteria Checklist)

- Negative
- Neutral
- Positive

Q44 References in reference list that should be reviewed and considered for inclusion:

________________________________________________________________

Q45 Comments for research team:

________________________________________________________________
